# Effects of protein intake from an energy-restricted diet on the skeletal muscle composition of overweight and obese rats

**DOI:** 10.1038/s41598-022-24961-5

**Published:** 2022-11-27

**Authors:** Ying Tian, Yuping Huan, Lihong Chen, Suwen Peng, Zhiyan He, Qian Wang

**Affiliations:** 1 Department of Nutrition and Food Hygiene, School of Public Health, Yangzhou University, Yangzhou, China; 2 Department of Cuisine and Nutrition, School of Tourism and Cuisine, Yangzhou University, Yangzhou, China; 3 Qingdao Eighth People’s Hospital, Qingdao, China

**Keywords:** Nutrition, Weight management

## Abstract

Excess weight and obesity are often associated with ectopic adipose tissue accumulation in skeletal muscles. Intermuscular adipose tissue (IMAT) impairs muscle quality and reduces insulin-stimulated skeletal muscle glucose uptake. Although energy restriction and high protein intake can decrease IMAT, the effects and mechanisms of protein intake from an energy-restricted diet on protein and fat masses in skeletal muscle have received little attention. After establishing a diet-induced overweight and obese Sprague-Dawley rat model (half male and half female), rats were divided into five groups: normal control (NC; normal weight, general maintenance diet), model control (MC; overweight and obesity, high-fat diet), energy-restricted low protein (LP; overweight and obesity, 60% energy intake of NC, general maintenance diet), energy-restricted normal protein (NP; overweight and obesity, 60% energy intake of NC, high-protein diet 1), and energy-restricted high protein (HP; overweight and obesity, 60% energy intake of NC, high-protein diet 2). After 8 weeks, plasma and skeletal muscle (quadriceps femoris and gastrocnemius) samples were collected. Plasma levels of glucose, triglycerides, and hormones were analyzed, while contents of protein, fat, and factors associated with their synthesis and degradation were evaluated in skeletal muscles. Plasma concentrations of hormones contrasted protein and fat contents in skeletal muscles. Fat weights and contents of quadriceps femoris and gastrocnemius muscles in the NP group were significantly lower compared with LP and HP groups (*P* < 0.05). Moreover, concentrations of factors associated with the degradation of muscle fat were significantly higher in the NP group compared with LP and HP groups (*P* < 0.05). During energy restriction, protein intake equal to that of a normal protein diet increased lipolysis of quadriceps femoris and gastrocnemius muscles in rats of both sexes.

## Introduction

Excessive energy intake is strongly associated with the current prevalence of overweight and obesity^1,2^. Excess weight and obesity are usually associated with ectopic adipose tissue accumulation, and skeletal muscle is one of the locations where adipose tissue accumulates. Intermuscular adipose tissue (IMAT) impairs muscle quality and reduces insulin-stimulated skeletal muscle glucose uptake^3,4^. Although energy restriction can decrease IMAT, unreasonable energy restriction will lead to the loss of lean body mass, including skeletal muscle mass, which can increase the risk of sarcopenia, especially in older adults^5,6^. Thus, an ideal weight loss strategy promotes satiety, maintains lean body mass, and reduces fat mass despite a negative energy balance^7^.

One potentially effective dietary strategy for decreasing IMAT in obese adults is increasing total protein intake^8^. As high-protein diets can suppress appetite, increase diet-induced thermogenesis, and help preserve lean body mass, they are recommended and promoted for overweight and obese adults^9,10^. A high-protein diet is characterized by increased intake of foods rich in protein. In energy-restricted high-protein diets for human weight loss, 25–30% of the energy is supplied by protein^9^. Although percentages of energy obtained from protein with a diet are higher than those of a normal-protein diet, high-protein diets advocate 0.8–1.2 g/kg of body weight per day, similar to a normal diet in terms of neutral energy balance^11,12^. The absolute amount of protein consumption was found to be of greater importance than the percentage of energy for weight loss management^13^, so actual protein intake during weight loss through energy restriction is more instructive for overweight or obese individuals.

Skeletal muscle is mainly made up of protein, accounting for 60% of the total body protein in humans^14,15^. Skeletal muscle is also a major site for fatty acid disposal^16^. Weight loss, achieved through energy-restricted diets, decreases both protein and fat mass in skeletal muscle^17,18^. However, the effects and mechanisms of protein intake from energy-restricted diets on protein and fat masses in skeletal muscle have received little attention.

In the present study, we assessed the effects of varying protein intake from an energy-restricted diet on the skeletal muscle composition (i.e. protein and fat contents in the quadriceps femoris and gastrocnemius) of overweight and obese rats of both sexes. Moreover, we explored the underlying mechanisms by which protein intake affected the synthesis and decomposition of protein and fat in these two types of skeletal muscle.

## Materials and methods

### Animals and diets

**Figure 1:**
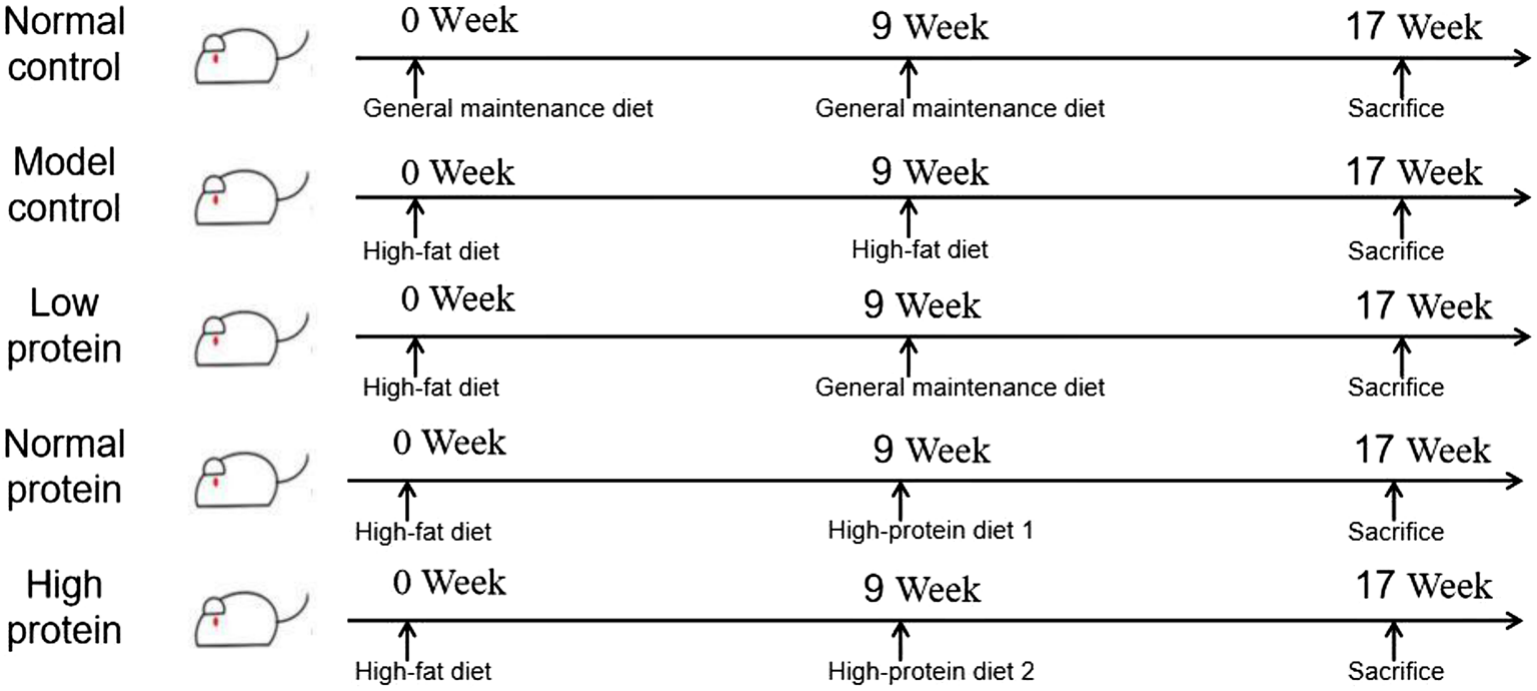
Experimental grouping schedule.

Seventy-two Sprague-Dawley rats (aged 6 weeks; half male and half female, weighing 190 ± 5 g and 160 ± 5 g, respectively) were obtained from Sippr-BK Laboratory Animals (Shanghai, China). Rats were housed three per cage in a controlled environment at 22 ± 2 °C and relative air humidity of 55% ± 15%, with a 12-h light–dark cycle. Six male rats and six female rats were randomly selected as two normal controls (NC), which were fed a general maintenance diet^19^. The other rats were fed high-fat diet for 9 weeks. At the end of the ninth week, rats whose body weight was more than 20% of the average body weight of the NC group were considered “obese”, while those whose body weight was 10–20% of the average body weight of the NC group were considered “overweight”. Overweight and obese rats were randomly divided into eight groups according to sex (n = 6/group): male and female rats in model control (MC) groups were fed a high-fat diet, males and females in energy-restricted low protein (LP) groups were fed a general maintenance diet, males and females in energy-restricted normal protein (NP) groups were fed high-protein diet 1, and males and females in energy-restricted high protein (HP) groups were fed high-protein diet 2 (Fig. 1). The compositions of diets are shown in Table 1. Protein contents of diets were determined by Kjeldahl analysis, fat contents were measured by Soxhelt extraction, contents of water and ash were detected by the weight method, and carbohydrate contents were determined by the subtraction method. The energy contents of diets were calculated according to the formula (1):

1 $$ {\text{Energy}}\,{\text{content}}\,\left( {{\text{kcal}}/{1}00\,{\text{g}}} \right)\, = \,\left[ {{\text{protein content}}\,\left( {{\text{g}}/{1}00{\text{ g}}} \right)\, \times \,{4}\,\left( {{\text{kcal}}/{\text{g}}} \right)} \right]\, + \,\left[ {{\text{fat}}\,{\text{content}}\,\left( {{\text{g}}/{1}00{\text{ g}}} \right)\, \times \,{9}\,\left( {{\text{kcal}}/{\text{g}}} \right)} \right]\, + \,\left[ {{\text{carbohydrate}}\,{\text{content}}\,\left( {{\text{g}}/{1}00{\text{ g}}} \right)\, \times \,{4}\,\left( {{\text{kcal}}/{\text{g}}} \right)} \right]. $$

**Table 1:** Compositions of the rat diets (g/100 g diet).

	High-fat diet	High-protein diet 1	High-protein diet 2
General maintenance diet^a^	42.0	78.5	30.0
Lard	28.0	–	–
Vegetable oil	–	1.5	4.0
Sucrose	10.0	–	–
Whole milk power	10.0	–	–
Soy isolate protein	8.0	18.0	61.0
Calcium hydrophosphate	2.0	1.0	2.5
Microcrystalline cellulose	–	1.0	2.5

^a^The compositions of the general maintenance diet were: corn 40.6%, middling 14.0%, wheat 10.0%, clover 3.0%, soybean meal 11.0%, fish meal 6.0%, chicken meal 6.0%, a mixture of vitamins and minerals 4.4%, wheat gluten 2.0%, stone power for feed 1.0% and salad oil 2.0%.

Rats in NC and MC groups had free access to food and water. Energy intake of LP, NP, and HP groups was 60% of that of the NC group. Protein intake percentages of LP, NP, and HP groups were 60%, 100%, and 200% of the NC group, respectively. Energy intake of the NC group was calculated on the first day, and dietary intake of LP, NP, and HP groups were calculated on the second day according to the formula (2):

2 $$ {\text{I}}_{{{\text{intervention}}}} \,\left( {\text{g}} \right)\, = \,\left[ {{6}0\% \, \times \,{\text{I}}_{{{\text{NC}}}} \,\left( {\text{g}} \right)\, \times \,{\text{EC}}_{{{\text{NC}}}} \,\left( {{\text{kcal}}/{\text{g}}} \right)} \right]\,/\,\left[ {{\text{EC}}_{{{\text{intervention}}}} \,\left( {{\text{kcal}}/{\text{g}}} \right)} \right] , $$

where I_intervention_ indicates dietary intake of the LP, NP or HP group; I_NC_ indicates dietary intake of the NC group; EC_NC_ indicates the energy content of the NC group diet; and EC_intervention_ indicates the energy content of the LP, NP, or HP group diet.

Body weights were measured weekly. At the end of the seventeenth week, after fasting for 12 h, all rats were sedated with sodium pentobarbital and sacrificed by abdominal aorta puncture to collect blood. Plasma was separated by low-speed centrifugation and stored at − 80 °C. The quadriceps femoris and gastrocnemius muscles were surgically excised. Subcutaneous fat in the groin and axilla, visceral fat in the mesentery and retroperitoneum, and fat around the kidneys and epididymis were all isolated. All tissues were weighed, immediately frozen in liquid nitrogen, and then stored at − 80 °C for subsequent analyses.

### Histological staining

Hematoxylin and eosin staining (H-E staining) was used to observe muscle structures. Muscles were fixed with 4% paraformaldehyde for 24 h, dehydrated in a graded series of alcohol (75%, 85%, 90%, 95%, and 100%), embedded in paraffin, sliced (4-µm-thick sections), deparaffinized, hydrated, and stained with hematoxylin and eosin. Distributions of adipose and muscle tissues were observed using a microscope (Nikon, Tokyo, Japan). Five fields were randomly selected in each sample for analyzing muscles, and three samples from each group were selected for statistical analysis. Image J software was used to analyze the percentage of fat in the muscles.

### Contents of protein and fat in skeletal muscles

The protein contents of quadriceps femoris and gastrocnemius muscles of rats were determined by Kjeldahl analysis^20^, and fat contents were measured by Soxhelt extraction^21^.

### Plasma parameters

Plasma concentrations of glucose and triglycerides were detected with a Hitachi 7020 automatic biochemical analyzer (Hitachi High-tech, Tokyo, Japan). Plasma concentrations of hormones related to the regulation of blood glucose and metabolism of skeletal muscles [i.e. insulin, glucagon, estradiol, testosterone, nitric oxide synthase (NOS), insulin-like growth factor-1 (IGF-1), growth hormone, and cortisol] were measured using ELISA kits according to the manufacturer’s instructions (Catalog Nos. HB127-Ra, HB112-Ra, HB898-Ra, HB779-Ra, HB131-Ra, HB124-Ra, HB364-Ra and HB459-Ra, respectively; R&D Systems, Minneapolis, MN, USA). ELISAs were based on the “double antibody sandwich” principle in which two highly specific antibodies are used to detect the target analyte. Plasma concentrations were calculated based on standard curves. Indeterminate results were excluded, and corresponding samples were retested.

### Skeletal muscle parameters

The concentrations of factors related to the synthesis and degradation of skeletal muscle protein and fat were measured in quadriceps femoris and gastrocnemius muscles using ELISA kits according to the manufacturer’s instructions (R&D Systems). Factors associated with the synthesis of skeletal muscle proteins included IGF-1 (Catalog No. HB124-Ra), phosphorylated phosphoinositide 3-kinase (p-PI3K, Catalog No. HB1281-Ra), phosphorylated Akt (p-Akt, Catalog No. HB534-Ra), phosphorylated mammalian target of rapamycin (p-mTOR, Catalog No. HB1282-Ra), and phosphorylated ribosomal protein S6 kinase 1 (p-S6K1, Catalog No. HB1283-Ra). Factors associated with the degradation of skeletal muscle protein included muscle atrophy F-box (MAFbx, Catalog No. HB1284-Ra) and muscle RING-finger protein-1 (MuRF-1, Catalog No. HB1285-Ra). Factors associated with the synthesis of skeletal muscle fat included nuclear sterol regulatory element binding protein-1C (nSREBP-1C, Catalog No. HB1288-Ra), fatty acid synthase (FAS, Catalog No. HB058-Ra), acetyl-CoA carboxylase (ACC, Catalog No. HB1289-Ra), stearoyl CoA desaturase-1 (SCD1, Catalog No. HB091-Ra), and lipin 1 (Catalog No. HB1290-Ra). Factors associated with the oxidation of skeletal muscle fat included phosphorylated adenosine monophosphate-activated protein kinase (p-AMPK, Catalog No. HB1291-Ra), peroxisome proliferator-activated receptor α (PPARα, Catalog No. HB735-Ra), and peroxisome proliferator-activated receptor γ (PPARγ, Catalog No. HB733-Ra).

### Statistical analysis

All quantitative data are presented as the mean ± standard deviation for variables with a normal distribution. One-way analysis of variance was used. If a significant difference (*P* < 0.05) was observed, post hoc analyses were conducted using the least-square difference approach. Data were analyzed using SPSS version 20 (IBM, Armonk, NY, USA).

### Ethical approval and consent to participate

All protocols in this study were approved by the Ethical Committee for Laboratory Animals of Yangzhou University (No. 201809.001), in accordance with ARRIVE guidelines^22^. All procedures and animal care were approved by the Ethical Committee for Laboratory Animals of Yangzhou University (No. 201809.001) and performed in accordance with relevant national and international guidelines, and the Guide for the Care and Use of Laboratory Animals published by the US National Institutes of Health.

## Results

### Contents of main nutrients and energy in rat diets

Table 2 shows the contents of protein, fat, carbohydrate, water, ash, and energy in each of the four diets. Fat and energy contents in the high-fat diet were higher than those in the general maintenance diet, and protein contents in the two high-protein diets were higher than the level in the general maintenance diet. Carbohydrate contents in the high-fat diet and both high-protein diets were lower than the level in the general maintenance diet.

**Table 2:** Contents of main nutrients and energy in each diet.

	Protein (g/100 g)	Fat (g/100 g)	Water (g/100 g)	Ash (g/100 g)	Carbohydrate (g/100 g)	Energy (kcal/100 g)
General maintenance diet	19.20	5.00	9.30	5.90	60.60	364.20
High-fat diet	19.11	29.26	8.20	5.29	38.14	492.34
High-protein diet 1	31.18	5.91	10.10	6.26	46.55	364.11
High-protein diet 2	54.90	5.83	10.30	6.54	22.43	361.79

### Body weight and body fat of rats

There were no significant differences between initial body weights of rats in NC and MC groups (*P* > 0.05). After modeling, body weights of rats in the MC group were significantly higher compared with the NC group (*P* < 0.05). Among these rats, 14 were overweight male rats (513.60 ± 16.21 g), 13 were overweight female rats (270.17 ± 6.00 g), 10 were obese male rats (596.40 ± 40.02 g), and 11 were obese female rats (294.13 ± 8.39 g). The final body weights of rats LP, NP, and HP groups were all significantly lower than rats in NC and MC groups (*P* < 0.05), but there were no significant differences in weight between the three energy-restricted groups (*P* > 0.05). The body weights of female rats were significantly lower than male rats (*P* < 0.05). Trends for body fat were consistent with those for body weight (Table 3).

**Table 3:** Body weight and body fat of rats in different groups (g).

	Male				Female			
	Initial body weight	Body weight after modeling	Final body weight	Final body fat	Initial body weight	Body weight after modeling	Final body weight	Final body fat
NC	195.06 $\pm$ 10.67^a^	462.80 $\pm$ 16.60^b^	554.60 $\pm$ 24.09^b^	27.91 ± 4.52^b^	164.83 $\pm$ 14.40^a^	241.58 $\pm$ 19.47^b^	275.18 $\pm$ 19.67^b^	10.30 ± 4.26^b^
MC	195.97 $\pm$ 8.19^a^	544.65 $\pm$ 49.05^a^	664.95 $\pm$ 90.97^a^	47.14 ± 11.67^a^	164.11 $\pm$ 7.92^a^	281.19 $\pm$ 13.79^a^	299.80 $\pm$ 19.75^a^	16.84 ± 3.32^a^
LP	–	–	470.80 $\pm$ 47.73^c^	14.64 ± 4.10^c^	–	–	234.67 $\pm$ 18.28^c^	2.06 ± 0.88^c^
NP	–	–	476.80 $\pm$ 42.51^c^	15.77 ± 6.69^c^	–	–	231.48 $\pm$ 11.87^c^	3.90 ± 1.80^c^
HP	–	–	458.45 $\pm$ 21.14^c^	12.54 ± 1.13^c^	–	–	230.47 $\pm$ 11.82^c^	3.55 ± 1.14^c^

*NC* normal control group, *MC* model control group, *LP* low energy low protein group, *NP* low energy normal protein group, *HP* low energy high protein group. Values are the mean ± SD. Mean values within a column with different superscript letters (a, b, c) were significantly different (*P* < 0.05).

### Dietary, energy and macronutrient intakes and percentages of energy obtained from macronutrients

Dietary, energy, and fat intake values of LP, NP, and HP groups were significantly lower compared with MC and NC groups (*P* < 0.05). With increased protein intake, the percentage of energy from protein in LP, NP, and HP groups increased and the intake of carbohydrate and percentage of energy from carbohydrate in these three groups decreased. Moreover, dietary, energy, and macronutrient intake values of female rats were significantly lower than those of male rats (*P* < 0.05) (Table 4).

**Table 4:** Intakes of diets, energy, macronutrients and the percentage of energy from macronutrients of rats in different groups.

		Diet intake (g/d)	Energy intake (kcal/d)	Protein intake (g/d)	Fat intake (g/d)	Carbohydrate intake (g/d)	Percentage of energy from protein (%)	Percentage of energy from fat (%)	Percentage of energy from carbohydrate (%)
Male	NC	24.06 $\pm$ 0.83^a^	87.63 $\pm$ 3.04^a^	4.62 $\pm$ 0.16^a^	1.20 $\pm$ 0.04^a^	14.58 $\pm$ 0.51^a^	21.09	12.36	66.56
	MC	21.57 $\pm$ 3.30^a^	112.19 $\pm$ 13.99^b^	4.12 $\pm$ 0.63^a^	6.31 $\pm$ 0.97^b^	8.23 $\pm$ 1.26^b^	15.53	53.49	30.99
	LP	14.46 $\pm$ 0.51^b^	52.66 $\pm$ 1.86^c^	2.78 $\pm$ 0.10^b^	0.72 $\pm$ 0.03^c^	8.76 $\pm$ 0.31^b^	21.09	12.36	66.56
	NP	14.47 $\pm$ 0.51^b^	52.70 $\pm$ 1.84^c^	4.51 $\pm$ 0.16^a^	0.86 $\pm$ 0.03^d^	6.74 $\pm$ 0.24^d^	34.25	14.61	51.14
	HP	14.56 $\pm$ 0.50^b^	52.68 $\pm$ 1.82^c^	7.99 $\pm$ 0.28^c^	0.85 $\pm$ 0.03^d^	3.27 $\pm$ 0.11^c^	60.70	14.50	24.80
Female	NC	14.80 $\pm$ 0.12^a^	53.91 $\pm$ 0.45^a^	2.85 $\pm$ 0.02^a^	0.74 $\pm$ 0.01^a^	9.36 $\pm$ 0.38^a^	21.09	12.36	66.56
	MC	11.82 $\pm$ 0.63^b^	58.18 $\pm$ 3.10^b^	2.26 $\pm$ 0.12^b^	3.46 $\pm$ 0.18^b^	4.07 $\pm$ 0.41^b^	15.53	53.49	30.99
	LP	9.28 $\pm$ 0.38^c^	33.78 $\pm$ 1.39^c^	1.78 $\pm$ 0.07^b^	0.46 $\pm$ 0.02^c^	5.62 $\pm$ 0.23^c^	21.09	12.36	66.56
	NP	9.29 $\pm$ 0.38^c^	33.83 $\pm$ 1.40^c^	2.90 $\pm$ 0.12^a^	0.55 $\pm$ 0.02^d^	4.33 $\pm$ 0.18^b^	34.25	14.61	51.14
	HP	9.34 $\pm$ 0.38^c^	33.78 $\pm$ 1.39^c^	5.13 $\pm$ 0.21^c^	0.54 $\pm$ 0.02^d^	2.09 $\pm$ 0.09^d^	60.70	14.50	24.80

*NC* normal control group, *MC* model control group, *LP* low energy low protein group, *NP* low energy normal protein group, *HP* low energy high protein group.

Values are the mean ± SD. Mean values of the same sex within a column with different superscript letters (a, b, c, d) were significantly different (*P* < 0.05).

### Histological staining

**Figure 2:**
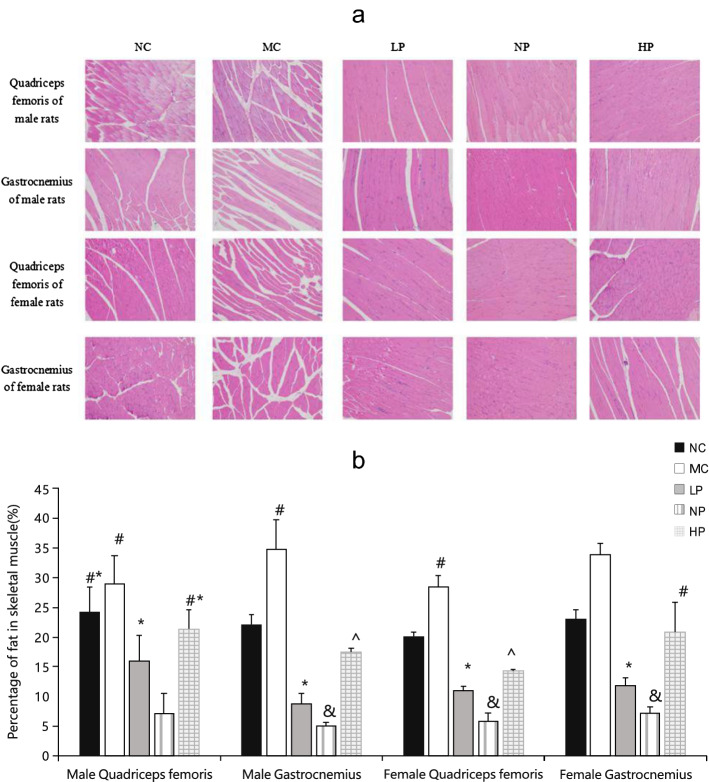
Histological analysis of the skeletal muscles. (**a**) The H-E staining of quadriceps femoris and gastrocnemius muscles of rats (100 ×). (**b**) Statistical analysis of fat in skeletal muscles. Mean values of the same sex with different superscript symbols (#,*, & and ^) were significantly different (*P* < 0.05).

As shown in Fig. 2, significantly more fat accumulated around skeletal muscle fibers in the MC group compared with the other groups. Moreover, significantly less fat was observed around skeletal muscle fibers of rats in NP group compared with the other groups.

### Composition of rat skeletal muscles

The weights of quadriceps femoris and gastrocnemius muscles, as well as weights and contents of protein and fat in these two muscles, were significantly lower in LP, NP, and HP groups compared with the MC group (*P* < 0.05). Because the gastrocnemius muscles of female rats were too lean, determination of fat contents in the female gastrocnemius was not possible. The weights and contents of fat in quadriceps femoris and gastrocnemius muscles of rats in the NP group were significantly lower compared with LP and HP groups (*P* < 0.05). There were no significant differences in protein contents in the quadriceps femoris muscle between male and female rats (*P* > 0.05), but all other data for female rats was significantly lower than that of male rats (*P* < 0.05) (Table 5).

**Table 5:** Weight and composition of quadriceps and gastrocnemius in different groups.

		Muscle weight (g)	Protein weight (g)	Fat weight (mg)	Protein content in muscle (mg/g)	Fat content in muscle (mg/g)
Quadriceps femoris	NC	12.70 $\pm$ 1.23^a,c^	2.64 $\pm$ 0.34^a^	83.71 ± 8.93^a^	207.82 $\pm$ 10.29^a^	6.59 ± 0.70^b^
	MC	14.44 $\pm$ 1.57^a^	4.61 $\pm$ 0.33^b^	98.27 ± 3.52^b^	321.97 $\pm$ 41.65^b^	6.81 ± 0.24^a^
	LP	12.28 $\pm$ 1.62^c^	3.29 $\pm$ 0.37^c^	28.86 ± 1.49^c^	268.50 $\pm$ 11.33^c^	2.35 ± 0.12^c^
	NP	10.23 $\pm$ 0.78^b^	2.59 $\pm$ 0.10^a^	18.00 ± 0.96^d^	254.29 $\pm$ 14.01^c^	1.76 ± 0.09^d^
	HP	10.24 $\pm$ 1.03^b^	2.50 $\pm$ 0.26^a^	21.91 ± 0.23^c^	245.41 $\pm$ 23.62^c^	2.14 ± 0.02^c^
**Male**						
Gastrocnemius	NC	5.81 $\pm$ 0.60^a,b^	1.21 $\pm$ 0.09^a^	8.29 $\pm$ 0.77^a^	201.40 $\pm$ 10.66^a^	1.38 $\pm$ 0.13^b^
	MC	6.01 $\pm$ 0.10^a^	1.89 $\pm$ 0.14^b^	9.95 $\pm$ 1.17^b^	327.00 $\pm$ 28.38^b^	1.71 $\pm$ 0.20^a^
	LP	5.38 $\pm$ 0.38^b^	1.23 $\pm$ 0.12^a^	3.47 $\pm$ 0.15^c^	226.78 $\pm$ 10.00^c^	0.63 $\pm$ 0.03^c^
	NP	5.31 $\pm$ 0.46^b^	1.38 $\pm$ 0.16^a^	2.43 $\pm$ 0.27^d^	260.59 $\pm$ 13.17^c^	0.46 $\pm$ 0.05^d^
	HP	5.40 $\pm$ 0.07^b^	1.25 $\pm$ 0.09^a^	3.12 $\pm$ 0.11^c^	237.55 $\pm$ 23.62^c^	0.58 $\pm$ 0.02^c^
Quadriceps femoris	NC	6.47 $\pm$ 0.31^a,b^	1.36 $\pm$ 0.15^a^	15.99 $\pm$ 1.23^a^	210.09 $\pm$ 23.62^a^	2.47 $\pm$ 0.19^a^
	MC	7.06 $\pm$ 0.61^a^	2.23 $\pm$ 0.17^b^	28.83 $\pm$ 2.35^b^	320.85 $\pm$ 21.66^b^	4.08 $\pm$ 0.33^b^
	LP	6.17 $\pm$ 0.44^b^	1.60 $\pm$ 0.14^a^	8.67 $\pm$ 0.45^c^	260.42 $\pm$ 23.64^c^	1.42 $\pm$ 0.07^c^
	NP	6.02 $\pm$ 0.48^b^	1.51 $\pm$ 0.21^a^	7.37 $\pm$ 0.26^d^	249.73 $\pm$ 24.65^c^	1.23 $\pm$ 0.04^d^
	HP	6.03 $\pm$ 0.65^b^	1.48 $\pm$ 0.24^a^	8.32 $\pm$ 0.19^c^	243.51 $\pm$ 23.21^c^	1.38 $\pm$ 0.03^c^
**Female**						
Gastrocnemius	NC	3.48 $\pm$ 0.21^a,c^	0.72 $\pm$ 0.04^a^	–	206.01 $\pm$ 12.54^a^	–
	MC	3.72 $\pm$ 0.43^a^	1.21 $\pm$ 0.19^b^	–	335.33 $\pm$ 27.82^b^	–
	LP	3.15 $\pm$ 0.32^c,d^	0.78 $\pm$ 0.10^a^	–	248.33 $\pm$ 26.22^c^	–
	NP	3.04 $\pm 0.16$ ^b,d^	0.81 $\pm$ 0.07^a^	–	264.17 $\pm$ 25.54^c^	–
	HP	3.15 $\pm$ 0.19^c,d^	0.78 $\pm$ 0.06^a^	–	249.22 $\pm$ 18.80^c^	–

*NC* normal control group, *MC* model control group, *LP* low energy low protein group, *NP* low energy normal protein group, *HP* low energy high protein group. Values are the mean ± SD.

Mean values of the same muscle type and the same sex within a column with different superscript letters (a, b, c, d) were significantly different (*P* < 0.05). Female rats had too few gastrocnemius muscles, so fat determination was not possible.

### Plasma parameters

**Figure 3:**
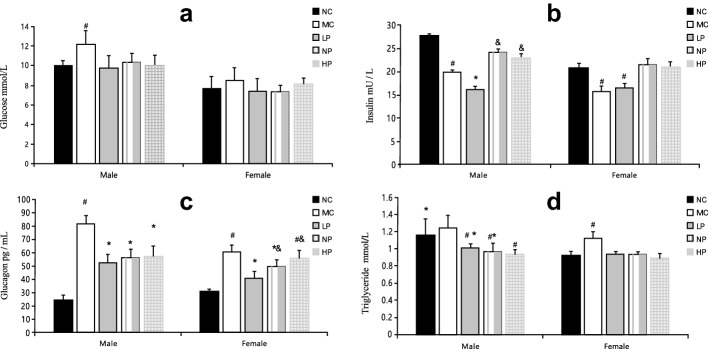
Concentration of glucose, insulin, glucagon and triglyceride in the plasma of different groups. (**a**) Statistical analysis of glucose. (**b**) Statistical analysis of insulin. (**c**) Statistical analysis of glucagon. (**d**) Statistical analysis of triglyceride. Mean values of the same sex with different superscript symbols (#, * and &) were significantly different (*P* < 0.05).

Figure 3 shows plasma concentrations of glucose, insulin, glucagon, and triglyceride in each group. For both male and female rats, triglyceride concentrations in LP, NP, and HP groups were significantly decreased compared with the MC group (*P* < 0.05); however, there was no significant difference in triglyceride levels among these three groups (*P* > 0.05). In contrast, plasma concentrations of glucose, insulin, and glucagon differed by sex. Plasma glucose concentrations of male LP, NP, and HP groups were significantly lower compared with the male MC group (*P* < 0.05), but female rats did not display a significant difference (*P* > 0.05). Plasma insulin concentrations were significantly increased in NP and HP groups compared with the MC group for both sexes (*P* < 0.05), but there was no significant difference in insulin concentration between NP and HP groups (*P* > 0.05). Glucagon concentrations of males in LP, NP, and HP groups were significantly lower than males in the MC group (*P* < 0.05), but there was no significant difference between female HP and MC groups (*P* > 0.05).

**Figure 4:**
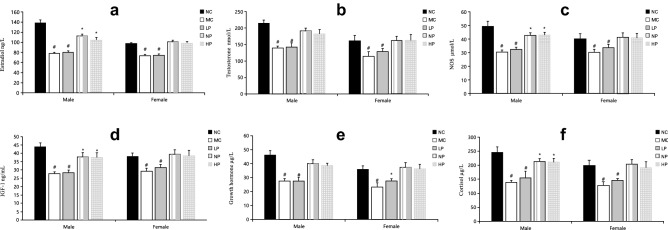
Concentration of hormones associated with the metabolism of skeletal muscle in the plasma of different groups. (**a**) Statistical analysis of estradiol. (**b**) Statistical analysis of testosterone. (**c**) Statistical analysis of NOS. (**d**) Statistical analysis of IGF-1. (**e**) Statistical analysis of growth hormone. (**f**) Statistical analysis of cortisol. Mean values of the same sex with different superscript symbols (#, *) were significantly different (*P* < 0.05).

Figure 4 shows plasma concentrations of hormones associated with skeletal muscle synthesis and degradation. The concentrations of all examined hormones were significantly lower in the MC group compared with the NC group (*P* < 0.05). Moreover, concentrations of hormones in NP and HP groups were significantly higher compared with the MC group (*P* < 0.05). However, there were no significant differences in hormone concentrations between NP and HP groups for either sex (*P* > 0.05).

### Skeletal muscle parameters

**Figure 5:**
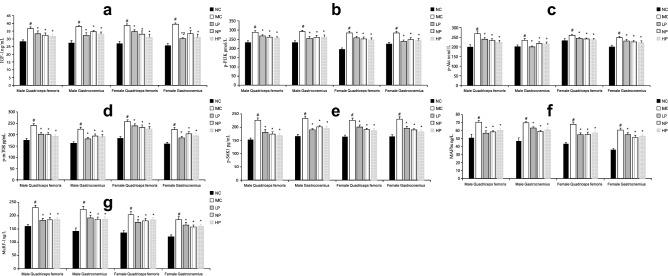
Concentration of factors in the pathways associated with the protein metabolism in the skeletal muscles of different groups. (**a**) Statistical analysis of IGF-1. (**b**) Statistical analysis of p-PI3K. (**c**) Statistical analysis of p-Akt. (**d**) Statistical analysis of p-mTOR. (**e**) Statistical analysis of p-S6K1. (**f**) Statistical analysis of MAFbx. (**g**) Statistical analysis of MuRF-1. Mean values of the same muscle type and the same sex with different superscript symbols (#, *) were significantly different (*P* < 0.05).

Figure 5 shows concentrations of factors associated with protein synthesis and degradation signaling pathways in the quadriceps femoris and gastrocnemius muscles. Concentrations of all factors were significantly increased in both muscles of the MC group compared with the NC group (*P* < 0.05). Moreover, concentrations of all factors were significantly lower in these two muscles in LP, NP, and HP groups compared with the MC group (*P* < 0.05). However, there were no significant differences among the three energy-restricted groups for either sex (*P* > 0.05).

**Figure 6:**
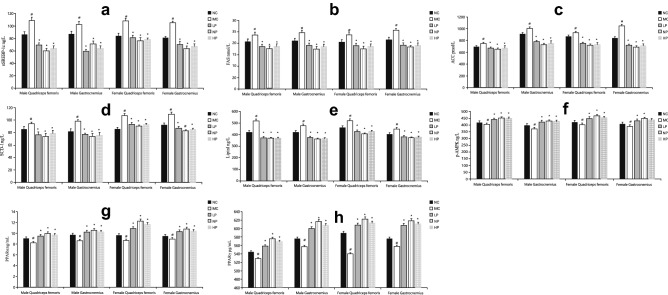
Concentration of factors in the pathways associated with the fat metabolism in the skeletal muscles of different groups. (**a**) Statistical analysis of nSREBP-1c. (**b**) Statistical analysis of FAS. (**c**) Statistical analysis of ACC. (**d**) Statistical analysis of SCD-1. (**e**) Statistical analysis of lipin1. (**f**) Statistical analysis of p-AMPK. (**g**) Statistical analysis of PPARα. (**h**) Statistical analysis of PPARγ. Mean values of the same muscle type and the same sex with different superscript symbols (#, *) were significantly different (*P* < 0.05).

Figure 6 shows concentrations of factors associated with fat synthesis and degradation in the quadriceps femoris and gastrocnemius muscles. Concentrations of all factors associated with the synthesis of skeletal muscle fat were significantly increased in quadriceps femoris and gastrocnemius muscles of the MC group compared with the NC group (*P* < 0.05). Moreover, concentrations of all these factors were significantly lower in these two muscles in LP, NP, and HP groups compared with the MC group (*P* < 0.05). However, there was no significant difference among the three energy-restricted groups for either sex (*P* > 0.05). In contrast, the concentrations of all factors associated with degradation of skeletal muscle fat in quadriceps femoris and gastrocnemius muscles of the MC group were significantly decreased compared with the NC group (*P* < 0.05), and significantly higher in LP, NP, and HP groups compared with the MC group (*P* < 0.05). In particular, the concentrations of factors associated with skeletal muscle fat degradation were significantly higher in the NP group compared with LP and HP groups for both sexes (*P* < 0.05).

## Discussion

Restricting energy intake is an effective way to lose weight. Intake of food for overweight or obese adults should be reduced by 30%–50% of energy intake from the habitual diet^9,23^. In the present study, the energy intake of rats in the three energy-restricted groups was reduced by 40% compared with the NC group, within the prescribed range. Protein intake of the LP group was reduced by 40% along with the reduction of energy intake, while protein intake of the NP group was the same as that of the NC group, and protein intake of the HP group was twice that of the NC group. Our results show that 40% energy restriction significantly reduced both total body weights and weights of the quadriceps femoris and gastrocnemius muscles, consistent with previous studies^9,24^. Furthermore, protein and fat contents in the quadriceps femoris and gastrocnemius muscles were significantly decreased in the three energy-restricted groups, consistent with previous studies^17,18^. However, the protein contents of muscles were not significantly higher in the HP group compared with LP and NP groups, potentially because carbohydrate intake in the HP group was only 20% of that in the NC group, and about 50% of that in the NP group. These results suggest that most protein intake in the HP group was used for gluconeogenesis to maintain plasma glucose levels, and there were not enough amino acids involved in skeletal muscle protein synthesis.

Excessive weight, obesity, and weight loss through energy restriction can lead to metabolic changes of glucose and lipid that may ultimate result in changes of glucose, insulin, glucagon, and triglyceride in the blood, most of which are “IMAT contributors”^8^. Table 5 shows that insulin concentrations were significantly decreased and glucagon concentrations were significantly increased in the plasma of male overweight and obese rats, resulting in significant increases of blood glucose. In contrast, glucose levels of female rats remained normal, potentially due to the smaller increase in glucagon in female overweight and obese rats compared with males. A previous study found that glucagon is secreted by α cells when energy demand is enhanced^25^. In the present study, blood was taken from rats after a 12-h fast, which led to an increased energy demand and subsequent stimulation of glucagon. Notably, saturated fatty acids more effectively stimulate glucagon release than unsaturated fatty acids^26^. The high-fat diet in the present study contained a lot of lard, which is rich in saturated fatty acids, so glucagon secretion of rats in the MC group was significantly higher compared with the other groups. Both high glucose and high free fatty acid can induce oxidative stress and endoplasmic reticulum stress in pancreatic β cells, which decreases their function to reduce insulin synthesis and secretion^27^. This was probably the reason why insulin concentrations in the MC group were significantly lower than the NC group for both sexes. When energy was restricted, insulin concentrations of NP and HP groups were significantly higher compared with the MC group, but there was no significant increase in the LP group. These results suggest that energy restriction alone did not improve β cell function and, instead, β cell function was only improved when energy restriction was accompanied by protein intake at or above a normal level. There was no significant difference in the increase of insulin between NP and HP groups, indicating that under energy restriction, even protein intake twice that of the normal level could not further increase insulin levels.

Skeletal muscle accounts for 30–45% of whole body protein metabolism and is a major site for fat disposal^28,29^. Thus, loss of skeletal muscle during weight loss through energy restriction may lead to metabolic changes of protein and fat, which can affect the composition of skeletal muscles. Endocrine and cellular mechanisms are two major factors affecting muscle composition^30^. The mechanisms responsible for changes in skeletal muscle composition during weight loss are related to changes in the synthesis and/or breakdown of protein and fat in skeletal muscles^5^. Some hormones present in the plasma may be involved in these processes. For example, insulin is responsible for protein and fat synthesis; estradiol, testosterone, NOS, IGF-1, and growth hormone are involved in protein synthesis; and cortisol can promote the breakdown of protein and fat^31,32^. In the present study, concentrations of all the aforementioned hormones were significantly increased in NP and HP groups compared with the MC group, in direct contrast to the protein and fat contents of skeletal muscles in these groups. These results suggest that hormones were probably not major factors affecting skeletal muscle composition during energy-restricted weight loss.

We next explored the mechanism by which skeletal muscle composition changes at the cellular level. In skeletal muscle cells, IGF-1 is a key factor stimulating protein synthesis. IGF-1 phosphorylates PI3Kl, which is followed by Akt phosphorylation and subsequent activation of mTOR, a key anabolic target. mTOR can activate S6K1 to promote protein translation^33,34^. In addition, the ubiquitin–proteasome system is a crucial protein degradation system in eukaryotes. MAFbx and MuRF1, two of the best-characterized E3 ubiquitin ligases in skeletal muscle, mediate polyubiquitination of proteins and target them to degradation by the proteasome^34^. In the present study, concentrations of factors in the IGF-1/PI3K/Akt/mTOR/S6K1 pathway, MAFbx, and MuRF1 were significantly decreased for both sexes in LP, NP, and HP groups compared with the MC group, and there was no significant difference among the three energy-restricted groups. These results suggest that energy restriction could reduce protein synthesis and breakdown in the skeletal muscles of both sexes, while dietary protein intake had no effect on protein metabolism in skeletal muscles when energy was restricted. In addition to the IGF-1/PI3K/Akt/mTOR/S6K1 pathway, several other signaling pathways are involved in protein synthesis, such as IGF-1/PI3K/Akt/mTOR/ eukaryotic initiation factor 4E (eIF4E), IGF-1/PI3K/Akt/glycogen synthase kinase 3β (GSK3β)/β-catenin, and IGF-1/PI3K/Akt/GSK3β/eIF2B pathways^34^. In the present study, energy restriction probably inactivated all IGF-1 related signaling pathways, resulting in a dominant reduction of protein synthesis in skeletal muscles that led to decreased protein contents of skeletal muscles in LP, NP, and HP groups.

Expression of SREBP-1c, which primarily activates genes required for the synthesis of fatty acids and triglycerides, is similar in human and mouse tissues, and highest in the adipose tissue of mice^35^. Previous studies showed that SREBP-1c could activate FAS, ACC, and SCD-1, the main enzymes in lipid synthesis^36^. Lipin-1 is an enzyme involved in de novo lipid synthesis through the glycerol-3-phosphate pathway^37^. Phosphorylation of AMPK, a metabolic master switch that regulates downstream signals based on shifts in the surrounding energy reservoir^38^, can decrease the activity of SREBP-1 and ACC to disrupt fatty acid synthesis^39^. However, AMPK can also activate PPAR-α and PPAR-γ^29,40^. PPAR-α is preferentially expressed in skeletal muscle, where it plays a key role in fatty acid catabolism^29^. PPAR-γ is expressed in both white adipose tissue, where it regulates cytokines production, and skeletal muscle, where it improves mitochondrial oxidative phosphorylation in skeletal muscle cells; accordingly, PPAR-γ can inhibit adipogenesis in both the IMAT and skeletal muscle cells^41,42^. In the current study, the concentrations of factors involved in fat synthesis were significantly decreased and those involved in fat decomposition were significantly increased in both sexes when energy was restricted. Changes of factors involved in the synthesis and decomposition of fat at both intermuscular and intramuscular levels were consistent with fat contents in the quadriceps femoris and gastrocnemius muscles, which were probably the main reason for the reduced fat content of skeletal muscles when energy intake was restricted in overweight and obese rats. Moreover, factors associated with the degradation of muscle fat were significantly higher in the NP group compared with LP and HP groups, consistent with lower fat contents in these two skeletal muscles in the NP group. Collectively, these results indicate that protein intake equal to a normal-protein diet could result in maximum breakdown of skeletal muscle fat when energy intake was restricted. These results also indicate that protein intake higher than that of a normal-protein diet did not more effectively reduce the fat contents of rat skeletal muscles.

## Conclusion

When energy intake was restricted, protein intake equal to a normal protein diet could result in higher breakdown of the fat in quadriceps femoris and gastrocnemius muscles of both male and female overweight and obese rats.

## Data Availability

The datasets generated or analyzed during the current study are available from the corresponding author upon reasonable request.

## Abbreviations

IMAT: Intermuscular adipose tissue
NOS: Nitric oxide synthase
IGF-1: Insulin-like growth factor-1
PI3K: Phosphoinositide 3-kinase
mTOR: Mammalian target of rapamycin
S6K1: Ribosomal protein S6 kinase 1
MAFbx: Muscle atrophy F-box
MuRF-1: Muscle RING-finger protein-1
SREBP-1C: Sterol regulatory element binding protein-1C
FAS: Fatty acid synthase
ACC: Acetyl-CoA carboxylase
SCD1: Stearoyl CoA desaturase-1
AMPK: Adenosine monophosphate-activated protein kinase
PPARα: Peroxisome proliferator-activated receptor α
PPARγ: Peroxisome proliferator-activated receptor γ

## References

[1] Nepocatych S, Melson CE, Madzima TA, Balilionis G (2019). Comparison of the effects of a liquid breakfast meal with varying doses of plant-based soy protein on appetite profile, energy metabolism and intake. Appetite.

[2] Leidy HJ, Ortinau LC, Fau-Douglas SM, Douglas SM, Fau-Hoertel HA, Hoertel HA (2013). Beneficial effects of a higher-protein breakfast on the appetitive, hormonal, and neural signals controlling energy intake regulation in overweight/obese, “breakfast-skipping”, late-adolescent girls. Am. J. Clin. Nutr..

[3] Schmitz-Peiffer C (2000). Signalling aspects of insulin resistance in skeletal muscle: Mechanisms induced by lipid oversupply. Cell Signal.

[4] Kelley DE, Goodpaster BH (2001). Skeletal muscle triglyceride. An aspect of regional adiposity and insulin resistance. Diab. Care.

[5] Cava E, Yeat NC, Mittendorfer B (2017). Preserving healthy muscle during weight loss. Adv. Nutr..

[6] Miller SL, Wolfe RR (2008). The danger of weight loss in the elderly. J. Nutr. Health Aging.

[7] Pesta DH, Samuel VT (2014). A high-protein diet for reducing body fat: Mechanisms and possible caveats. Nutr. Metab..

[8] Wright CA-O, Zhou J, Sayer RA-O, Kim JE, Campbell WA-O (2018). Effects of a high-protein diet including whole eggs on muscle composition and indices of cardiometabolic health and systemic inflammation in older adults with overweight or obesity: A randomized controlled trial. Nutrients.

[9] Waliłko E, Napierała M, Bryśkiewicz M, Fronczyk A, Majkowska LA-O (2021). High-protein or low glycemic index diet-which energy-restricted diet is better to start a weight loss program?. Nutrients.

[10] Tang M, Armstrong CL, Fau-Leidy HJ, Leidy HJ, Fau-Campbell WW, Campbell WW (2013). Normal vs. high-protein weight loss diets in men: Effects on body composition and indices of metabolic syndrome. Obesity.

[11] Wycherley TP, Moran LJ, Fau-Clifton PM, Clifton PM, Fau-Noakes M, Noakes M, Fau-Brinkworth GD, Brinkworth GD (2012). Effects of energy-restricted high-protein, low-fat compared with standard-protein, low-fat diets: A meta-analysis of randomized controlled trials. Am. J. Clin. Nutr..

[12] Westerterp-Plantenga MS, Lemmens SG, Fau-Westerterp KR, Westerterp KR (2012). Dietary protein—Its role in satiety, energetics, weight loss and health. Br. J. Nutr..

[13] Westerterp-Plantenga MS, Nieuwenhuizen A, Fau-Tomé D, Tomé D, Fau-Soenen S, Soenen S, Fau-Westerterp KR, Westerterp KR (2009). Dietary protein, weight loss and weight maintenance. Annu. Rev. Nutr..

[14] Wagenmakers AJ (1998). Muscle amino acid metabolism at rest and during exercise: Role in human physiology and metabolism. Exerc. Sport Sci. Rev..

[15] Wagenmakers AJ (1999). Tracers to investigate protein and amino acid metabolism in human subjects. Proc. Nutr. Soc..

[16] Jeukendrup AE (2002). Regulation of fat metabolism in skeletal muscle. Ann. N. Y. Acad. Sci..

[17] Dulloo AG, Jacquet J, Fau-Girardier L, Girardier L (1996). Autoregulation of body composition during weight recovery in human: The Minnesota experiment revisited. Int. J. Obes. Relat. Metab. Disord..

[18] Elia M, Stubbs RJ, Fau-Henry CJ, Henry CJ (1999). Differences in fat, carbohydrate, and protein metabolism between lean and obese subjects undergoing total starvation. Obes. Res..

[19] Standardization Administration of China. Vol. GB 14924.3–2010 (China Standard Press, Beijing, China, 2010).

[20] Rizvi NB, Aleem S, Khan MR (2022). Quantitative estimation of protein in sprouts of Vigna radiate (Mung Beans), Lens culinaris (Lentils), and Cicer arietinum (Chickpeas) by Kjeldahl and lowry methods. Molecules.

[21] López-Bascón MA, de Castro MDL (2020). Handbooks in Separation Science.

[22] Kilkenny C, Browne W, Cuthill IC, Emerson M, Altman DR (2010). Animal research: reporting in vivo experiments: the ARRIVE guidelines. Brit. J. Pharmacol..

[23] Wang Y, Sun M, Yang Y (2019). China Blue Paper on Obesity Prevention and Control.

[24] Jovanovski E (2021). Effect of viscous fiber supplementation on obesity indicators in individuals consuming calorie-restricted diets: A systematic review and meta-analysis of randomized controlled trials. Eur. J. Nutr..

[25] Gerich JE, Langlois M, Schneider V, Karam JH, Noacco C (1974). Effects of alternations of plasma free fatty acid levels on pancreatic glucagon secretion in man. J. Clin. Investig..

[26] Müller TD, Finan B, Clemmensen C, DiMarchi RD, Tschöp MH (2017). The new biology and pharmacology of glucagon. Physiol. Rev..

[27] Hasnain SZ, Prins JB, McGuckin MA (2016). Oxidative and endoplasmic reticulum stress in β-cell dysfunction in diabetes. J. Mol. Endocrinol..

[28] Gasier HG, Fluckey JD, Previs SF (2010). The application of ^2^H_2_O to measure skeletal muscle protein synthesis. Nutr. Metab..

[29] Stephenson EJ (2012). Skeletal muscle respiratory capacity is enhanced in rats consuming an obesogenic Western diet. Am. J. Physiol. Endocrinol. Metab..

[30] Millward DA-O (2021). Interactions between growth of muscle and stature: Mechanisms involved and their nutritional sensitivity to dietary protein: The protein-stat revisited. Nutrients.

[31] Kraemer WJ, Ratamess NA, Hymer WC, Nindl BC, Fragala MS (2020). Growth hormone(s), testosterone, insulin-like growth factors, and cortisol: Roles and integration for cellular development and growth with exercise. Front. Endocrinol..

[32] Biagetti BA-O, Simó RA-O (2021). GH/IGF-1 abnormalities and muscle impairment: From basic research to clinical practice. Int. J. Mol. Sci..

[33] Timmer LT, Hoogaars WMH, Jaspers RT (2018). The role of IGF-1 signaling in skeletal muscle atrophy. Adv. Exp. Med. Biol..

[34] Yoshida TA-O, Delafontaine P (2020). Mechanisms of IGF-1-mediated regulation of skeletal muscle hypertrophy and atrophy. Cells.

[35] Engelking LJ, Cantoria MJ, Xu Y, Liang G (2018). Developmental and extrahepatic physiological functions of SREBP pathway genes in mice. Semin. Cell Dev. Biol..

[36] Ren LP (2016). Impact of activating transcription factor 4 signaling on lipogenesis in HepG2 cells. Mol. Med. Rep..

[37] Brohée LA-O, Crémer J, Colige A, Deroanne C (2021). Lipin-1, a versatile regulator of lipid homeostasis, is a potential target for fighting cancer. Int. J. Mol. Sci..

[38] Kim Y, Park CW (2019). Mechanisms of adiponectin action: Implication of adiponectin receptor agonism in diabetic kidney disease. Int. J. Mol. Sci..

[39] Li Y (2011). AMPK phosphorylates and inhibits SREBP activity to attenuate hepatic steatosis and atherosclerosis in diet-induced insulin-resistant mice. Cell Metab..

[40] Li G, Wang J, Ye J, Zhang Y, Ying Z (2015). PPARα protein expression was increased by four weeks of intermittent hypoxic training via AMPKα2-dependent manner in mouse skeletal muscle. PLoS ONE.

[41] Evans RM, Barish GD, Fau-Wang Y-X, Wang YX (2004). PPARs and the complex journey to obesity. Nat. Med..

[42] Bhattamisra SA-O, Koh HA-O, Lim SY, Choudhury H, Pandey MA-O (2021). Molecular and biochemical pathways of catalpol in alleviating diabetes mellitus and its complications. Biomolecules.

